# Simplified insecticide toxicity determination method for *Nesidiocoris tenuis* using contaminated diet

**DOI:** 10.1016/j.mex.2021.101220

**Published:** 2021-01-07

**Authors:** David Wari, Motonori Takagi, Takashi Ogawara

**Affiliations:** Horticultural Research Institute, Ibaraki Agricultural Center, Ago 3165-1, Kasama City, Ibaraki 319-0292, Japan

**Keywords:** Artemia cysts, Nesidiocoris tenuis, Toxicity assays

## Abstract

*Nesidiocoris tenuis* is a zoophytophagous mirid bug widely studied for its role in preying on major greenhouse pests. Since *N. tenuis* has now been known for its vigorous predation potential on pests such as *Bemisia tabaci*, many applied entomologists are now recommending that *N. tenuis* be merged into the Integrated Pest management (IPM) systems. However, successful integration of *N. tenuis* into any IPM system depends on thorough evaluation with compatible pesticides, as incompatible pesticides can offset the whole idea of IPM. Here, we simulate the field situation where *N. tenuis* feeds directly on a contaminated *B. tabaci* nymph or leaves. However, instead of using live *B. tabaci* nymphs, we used brine shrimp eggs, *Artemia salina* (Linnaeus, 1758). Brine shrimp eggs have been reported to be an excellent factitious supplementary diet in augmenting *N. tenuis* populations. Thus, we use brine shrimp eggs to determine the toxicity of pesticides, to which the calculated mortality rates can be used to determine which pesticides can be used together with *N. tenuis* in an IPM system against any related pest.

• We developed a customized containment system that promotes aeration and minimize contamination.
• Pesticide contaminated hatched brine shrimp eggs is delivered to *N. tenuis* in the aerated containment system.
• In addition to established methods such as leaf dipping or insect dipping, this method shows to mimic *N. tenuis* feeding on contaminated *B. tabaci* nymphs in field conditions thus, predicts how a pesticide may be of toxic or compatible with *N. tenuis* when both are integrated together.

## Graphical abstract

Schematic representation of the customized containment tube housing the *Nesidiocoris tenuis* with contaminated brine shrimp eggs.

**Figure:**
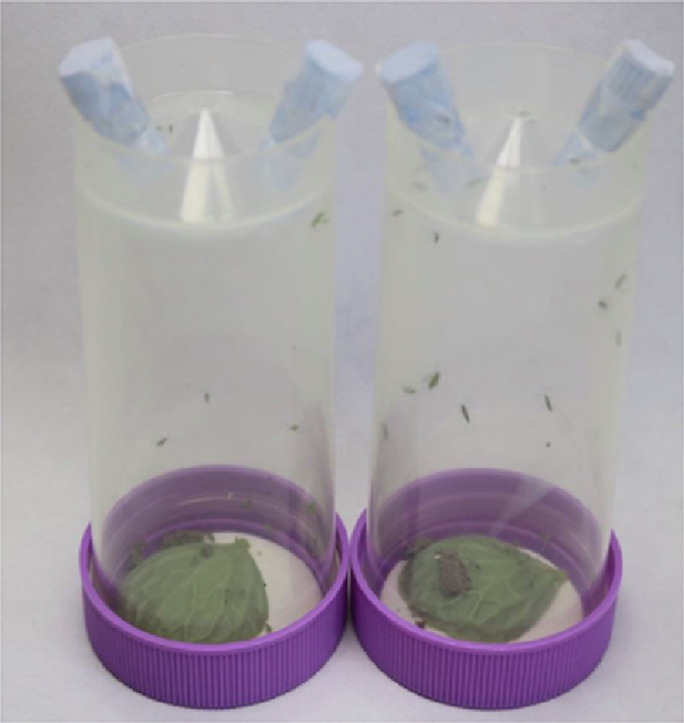

Specifications table

**Table:** 

Subject Area	Agricultural and Biological Sciences
More specific subject area	Biological Control
Method name	Insecticide toxicity determination using contaminated diet
Name and reference of original method	Evaluation of Predator Consumption of Contaminated eggs, Wanumen et al 2016 (doi: 10.1093/jisesa/iew084)
Resource availability	Non

**Reagents and Equipment Required**

Pesticides (Insecticides and Fungicides)

Brine Shrimp eggs (*Artemia salina* L., Agri-Soken Inc.)

50mL falcon tube (Iwaki AGC Techno Glass Co., Ltd)

15mL falcon tube (Iwaki AGC Techno Glass Co., Ltd)

Filter paper (Advantec, Toyo Roshi Kaisha, Ltd.)

Sodium Chloride (Junsei Chemical Co. Ltd.)

Magnesium Sulphate (Junsei Chemical Co. Ltd.)

Note: This list includes only non-standard items. Common laboratory equipment such as beaker, spatula, measuring cylinders are assumed to be available.

## Preparation

### Containment tube preparation

**Fig. 1:**
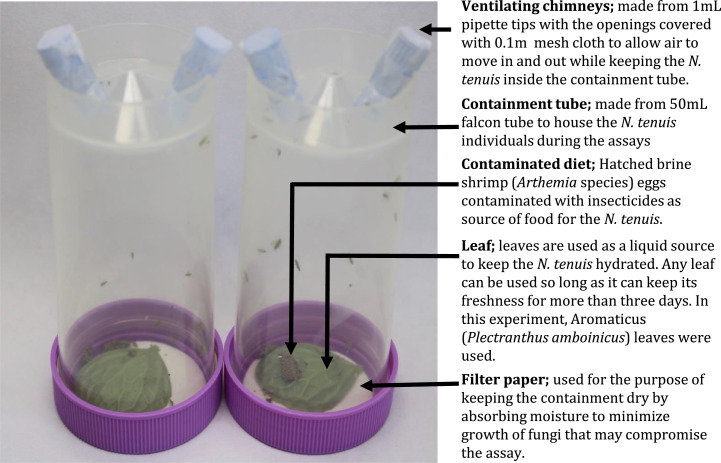
Schematic representation of the customized containment tube using a 50mL falcon tube and pipette tips to minimize contamination while improving aeration for *N. tenuis* comfortability that can promote feeding on brine shrimp eggs thus, ingestion of the contaminated diet.

In order to minimize outside influence and contamination, a containment tube needs to be prepared to house the biological control agents and, in this case, *Nesidiocoris tenuis* subjected to contaminated brine shrimp eggs and leaf (leaves from any plants that *N. tenui*s uses as host) as a source of liquid diet. To house 15 to 20 individuals of *N. tenuis* (be it nymphs or adults), a 50mL falcon tube is required. For ventilation, drill in two holes befitting the 1000µL pipette tip on the bottom side of the 50ml falcon tube. Fasten the protruding ends of the pipette tip shielded with a 0.1µm mass. The two pipette ends fastened with a 0.1µm mass should allow for air ventilation and not suffocate the live specimens inside the containment. The bottom side should now be the topside of the containment. The top side of the falcon tube (where the lid is) is now inverted and should be the bottom side (or the stand) for the containment. Since live specimens treated with pesticides are placed in the containment, fungal growth from the presence of moisture is expected. To avoid fungal growth, place a filter paper trimmed to fit the inside of the 50mL falcon tube lid. (See Fig. 1).

### Brine shrimp eggs preparation

Brine shrimp eggs, any species from the genus *Artemia* is ideal. Since brine shrimp eggs are covered with an outer shell that will be permeably hard and contamination with any pesticide is impossible, eggs must be hatched in order to be contaminated with any pesticide, thus, successful delivery of an active ingredient when ingested. To hatch the eggs, weigh 0.05 to 0.08g of brine shrimp and place the weighed eggs in a 15mL falcon tube. Add 10mL of sodium chloride (25 parts per thousand) with its pH adjusted to 8.0 using magnesium sulphate (this solution mimics the sea water used by brine shrimps when hatching their eggs). To allow aeration, drill a hole on the lid of the falcon tube then fasten. Place the 15mL falcon tube in an incubator with optimal temperature of 26–28°C and well illuminated (L16:D8hrs) to trigger hatching mechanism within the embryo. After 16–24h incubation, remove from the incubator and decant off the liquid and keep the hatched eggs. Confirm using a light microscope if the hatching has been successful. Before dipping into pesticide solutions, sterilize in a clean bench using a UV light for at least an hour to eliminate any contamination by microbes during the incubation period or confirmation using light microscope.

### Additional preparations

In addition to brine shrimp eggs as the pesticide contamination source, a liquid supplement source to support *N. tenuis* in addition to the eggs as food source is necessary. Any plant that is known to be a host to *N. tenuis* is sufficient enough for use during the assay. Leaves roughly of 40 mm in diameter that should sit precisely on the filter paper placed in the inner side of the 50mL falcon tube lid should be prepared as well.

## Procedure

1. Prepare appropriate dosage of pesticide solution in a beaker (in this case, 200mL beaker would be adequate to allow dipping of brine shrimp eggs and leaves to be used during the assay).
2. Leaf-dip the prepared leaves into the pesticide solutions for 25 s and air dry for 60–90 min
3. Similarly, after sterilization using UV light, dip the hatched brine shrimp eggs into the pesticide solution for 25 s and air dry for 60–90 min
4. After drying, first place the leaves on the filter paper within the lid of the 50mL falcon tube. Then add the hatched contaminated eggs onto the leaves.
5. Place 15-20 individuals of *N. tenuis*, it can be either nymphs only or adult only.
6. Place the containment containing the contaminated leaves and eggs with the *N. tenuis* in an incubator with laboratory conditions of 25±1°C, 65±10% RH and 16:8 L:D photoperiod.
7. After 72hrs, dead or alive individuals can be assessed by touching them with a brush. No movement is considered dead.
8. Mortality rate can be calculated using Abbotts formula [1] or the following simple formula for Corrected Mortality Rate (%) = [(control - test)/control] × 100%.

## Method validation

*Bemisia tabaci* is a notorious pest for major vegetable crops and until recently, *B. tabaci* management has been mostly relied upon insecticides as the primary control approach. Augmenting Biological Control Agents (BCAs); i.e. parasitoids, predators and microbial entomopathogenic fungi into the chemical control system offers a key strategy, whose potential have been realized however lacking comprehensive studies in open and/or protected cropping systems. To minimize the overuse of insecticides, augmenting *N. tenuis* has been promoted and recommended to be assimilated into *B. tabaci* Integrated Pest Management (IPM) system. However, to use both insecticides and *N. tenuis* in the *B. tabaci* IPM system, highly toxic insecticides must be avoided as much as possible in-order for the biological control agents such as *N. tenuis* to work efficiently. To determine insecticides that are toxic to *B. tabaci* but less toxic to *N. tenuis*, we developed a bioassay method using contaminated brine shrimp eggs as well as contaminated leaves to mimic *N. tenuis* feeding on live specimens of *B. tabaci* nymphs and tomato plants when sprayed upon with an insecticide. Using four insecticides registered for *B. tabaci* control in tomato production in Ibaraki Prefecture, Japan as representatives from insecticides use in *B. tabaci* control, bioassays were performed on nymphs and adults of *B. tabaci* and *N. tenuis*. Here, we show that this method, while simulating actual field situations, we used insecticide contaminated brine shrimp eggs to pre-determine the compatibility and incompatibility of insecticides against *N. tenuis* nymphs and adults.

Bioassay results revealed that dinotefuran, was toxic to both *B. tabaci* and *N. tenuis* nymphs and adults with more than 80% mortality (Table 1), suggesting that neonicotinoids are harmful and may not be compatible for use with *N. tenuis* in field conditions. Milbemectin, lepimectin and cyantraniliprole were observed to be toxic to *B. tabaci* nymphs and adults (corrected mortality equal to or greater than 70%) but less toxic to *N. tenuis* nymphs and adults (corrected mortality equal to or less than 70%) (Table 1). To further confirm these mortality rates, lethal dose (LD) was determined. Using the hatched contaminated brine shrimp eggs method, bioassays were performed and LD_50_ computed (See Table 2). LD_50_ results revealed that LD_50_ values for *N. tenuis* nymphs and adults against dinotefuran was 21 and 1,000 folds respectively lower to that of the agriculturally recommend dose, indicating that dinotefuran is highly toxic to *N. tenuis* nymphs and adults. This observation further verifies that dinotefuran and *N. tenuis* are incompatible to be used together. Milbemectin had LD_50_ values more than 29,300 and 200 folds higher than the agriculturally recommended dose for *N. tenuis* nymphs and adults, respectively, indicating that milbemectin is less toxic to *N. tenuis* nymphs and adults. Similarly, cyantraniliprole had higher LD_50_ values compared to its agriculturally recommended dose indicating less toxicity against *N. tenuis* nymphs and adults. Lepimectin, an insecticide with similar properties to that of milbemectin (ranked in the same IRAC code), had LD_50_ values 3.5 fold higher than the agriculturally recommended dose against *N. tenuis* nymphs, however, 4.2 folds lower against the adults compared to the agriculturally recommended dose. All insecticides used in this study considered, dinotefuran, a representative of neonicotinoids can be toxic to *N. tenuis*, therefore, incompatible to use together during *B. tabaci* management. Cyantraniliprole, an example of diamides can be less toxic to natural enemies such as *N. tenuis* but toxic to pests such as *B. tabaci*, thus, combining diamides and natural enemies may augment each other in managing pests in closed systems such as tomato crops in the vinyl or glass houses. Insecticides that belong to the avermectins and milbemycins varied in their toxicity against *N. tenuis* nymphs and adults but toxic to *B. tabaci* nymphs and adults.

**Table 1:** *Nesidiocoris tenuis* nymphs and adult corrected mortality (according to Abbott's formula) after ingestion of contaminated brine shrimp eggs compared to mortality rate of *Bemisia tabaci* nymphs and adults using leaf dipping method computed using Abbott's formula.

Insecticide	IRAC Code^1^	Agriculturally recommended dose	*B. tabaci* nymphs^2^		*B. tabaci* adults^2^		*N. tenuis* nymphs^3^		*N. tenuis* adults^3^	
			n^4^	Mortality (%)^5^	n	Mortality (%)	n	Mortality (%)	n	Mortality (%)
Dinotefuran	4A	0.5 mg ml^−1^	1645	90.6±2.7	246	98.7±1.3	47	82.1±4.0	49	90.0±14.4
Milbemectin	6	0.75 µl ml-1	2439	98.3±0.5	265	74.7±5.8	45	9.1±3.9	46	52.3±6.3
Lepimectin	6	0.5 µl ml-1	1883	98.2±0.3	251	94.9±2.7	62	4.9±3.3	49	20.7±16.3
Cyantraniliprole	28	0.5 µl ml^−1^	1118	93.9±3.2	226	82.5±7.0	42	26.0±21.4	44	37.7±20.3

1 IRAC codes are as per stipulated in the Insecticides Resistance Action Committee manual (https://irac-online.org/modes-of-action/), visited August 18, 2020

2 *B. tabaci* mortality data generated from leaf-dipping methods as described in our previous study, Wari et al. [2]

3 *N. tenuis* mortality data generated from ingestion of contaminated hatched brine shrimp eggs

4 number of individuals used in the toxicity assay

5 corrected mortality using Abbotts formula

**Table 2:** Lethal dosage toxicity assay for *Nesidiocoris tenuis* validating the dosage as recommended for field use as shown in Table 1.

Insecticide	IRAC Code^1^	Agriculturally recommended dose (ppm)^2^	Developmental stage	LD_50_ (ppm)	95% confidence interval	Standard deviation
Dinotefuran	4A	100	Nymphs	4.67	3.88 - 5.88	2.89
			Adults	0.10	0.08 - 0.13	3.71
Milbemectin	6	6.5	Nymphs	190,713.47	1,706.36 - 8.4 × 10^15^	21,728.33
			Adults	1,308.34	212.73 - 65820.47	287.95
Lepimectin	6	5	Nymphs	17.66	9.84 – 37.79	26.89
			Adults	1.18	0.68 – 2.04	38.56
Cyantraniliprole	28	51.5	Nymphs	353,073.19	1,275.20 - 6.83 × 10^9^	470.83
			Adults	47,448.72	1,890.83 - 1.50 × 10^10^	21628.89

1 IRAC codes are as per stipulated in the Insecticides Resistance Action Committee manual (https://irac-online.org/modes-of-action/), visited August 18, 2020

2 Agriculturally recommended dosage depicted in ppm. The active ingredient for each insecticides are as follows; Dinotefuran (1.0%), Milbemectin (1.0%), Lepimectin (1.0%) and Cyantraniliprole (20.0%)

In a previous report, *Ephestia kuehniella* (Lepidoptera:Pyralidae) eggs treated with six insecticides (flubendiamide, spirotetramat, deltamethrin, flonicamid, metaflumizone, and sulfoxaflor) were fed to *N. tenuis* under laboratory conditions for three days [3]. All six insecticides showed reduced mortality although, metaflumizone, sulfoxaflor, flonicamid and flubendiamide showed effects at sub-lethal dose affecting offspring production and longevity. Similarly, Planes et al. [4] showed that spirotetramat was harmless to *Cryptolaemus montrouzieri* Mulsant (Coleoptera: Coccinellidae), a natural enemy of *Planococcus citri* Risso (Hemiptera: Pseudococcidae) when *P. citri* was contaminated with spirotetramat and fed to *C. montrouzieri*. On the other hand, Lucas et al. [5] tested the toxicity of four insecticides (imidacloprid, cryolite, cyromazine and *Bacillus thuringiensis* var. *tenebrionis*) against 12-spotted lady beetle, *Coleomegilla maculata lengi* Timberlake (Coleoptera: Coccinellidae) by feeding it with insecticide contaminated Colorado potato beetle, *Leptinotarsa decemlineata* (Say) (Coleoptera: Chrysomelidae). Their results showed that exposure to prey (*L. decemkineata*) contaminated with imidacloprid (a known neonicotinoid) had negatively impacted the predator (*C. maculata*). All taken together, exposure of natural enemies to factitious diets or prey contaminated with insecticides with varying mode of actions showed varying effects on the mortality of natural enemies/predator. Therefore, careful consideration is paramount when augmenting natural enemies and the insecticides with different classes or mode of actions. Nevertheless, the underlying principle been that, compared to established methods, i.e. leaf dipping or topical applications [5], insecticide toxicity determination using factitious diet can effectively be authentic in determining the compatibility and incompatibility of insecticides with natural enemies. As different insecticides with different mode of actions have been in development, targeting different sites, organs and tissues, thus a need for a method that is specific but easy and practical to use are needed, and this method provides the avenue for such case. Moreover, since many natural enemies have shown prospects in using brine shrimp eggs as factitious dietary, accordingly, further the cause in using brine shrimp eggs as a medium for delivering insecticides to natural enemies to test for their compatibility.

## Declaration of Competing Interest

The authors declare that they have no known competing financial interests or personal relationships that could have appeared to influence the work reported in this paper.
